# CENPE and LDHA were potential prognostic biomarkers of chromophobe renal cell carcinoma

**DOI:** 10.1186/s40001-023-01449-0

**Published:** 2023-11-04

**Authors:** Hui-feng Wu, Hao Liu, Zhe-wei Zhang, Ji-min Chen

**Affiliations:** Department of Urology, The Second Affiliated Hospital, Zhejiang University School of Medicine, No. 88 Jiefang Road, Shangcheng District, Hangzhou, 310009 Zhejiang China

**Keywords:** CENPE, LDHA, Chromophobe renal cell carcinoma, Prognosis

## Abstract

**Background:**

Most sarcomatoid differentiated renal cell carcinoma was differentiated from Chromophobe renal cell carcinoma (KICH) and related to a bad prognosis. Thus, finding biomarkers is important for the therapy of KICH.

**Methods:**

The UCSC was used for determining the expression of mRNA and miRNA and clinical data in KICH and normal samples. KEGG and GO were used for predicting potential function of differently expressed genes (DEGs). Optimal prognostic markers were determined by Lasso regression. Kaplan–Meier survival, ROC, and cox regression were used for assessing prognosis value. GSEA was used for predicting potential function of markers. The relations between markers and immune cell infiltration were determined by Pearson method. The upstream miRNA of markers was predicted in TargetScan and DIANA.

**Results:**

The 6162 upregulated and 13,903 downregulated DEGs were identified in KICH. Further CENPE and LDHA were screened out as optimal prognostic risk signatures. CENPE was highly expressed while LDHA was lowly expressed in KICH samples, and the high expressions of 2 genes contributed to bad prognosis. The functions of CENPE and LDHA were mainly enriched in proliferation related pathways such as cell cycle and DNA replication. In addition, the correlation of 2 genes with immune infiltrates in KICH was also observed. Finally, we found that has-miR-577 was the common upstream of 2 genes and the binding sites can be predicted.

**Conclusion:**

CENPE and LDHA were identified as the important prognostic biomarkers in KICH, and they might be involved in the proliferation of cancer cell.

## Introduction

Chromophobe renal cell carcinoma (KICH) is a subtype of renal cell carcinoma (RCC) [1], which is first described in 1985 and accounts for 5% of RCC [2, 3]. The lack of rich vascular networks, which usually could be observed in clear-cell carcinomas, is a characteristic of KICH [4]. Numerous studies revealed that the prognosis of KICH is better than other subtypes of RCC [5]. In addition, the prognosis of most KICH was not related to the pathological stage, while the other subtypes were associated with the pathological stage [6]. However, KICH is the main kind of primary subtype of the sarcomatoid differentiated RCC, which is generally related to a bad prognosis [7]. Therefore, it is of critical importance to identify prognosis-related biomarkers, which are helpful for the diagnosis and treatment strategy selection of KICH.

Centrosome-associated protein E (CENPE) is a plus end-directed kinetochore motor protein, which accumulates at the G2 phase of the cell cycle and plays a vital role in mitosis [8, 9]. Previous studies confirmed that CENPE promoted the proliferation of multiple cancers, such as ovarian cancer, lung adenocarcinoma and lung adenocarcinoma [8, 10, 11]. Besides, Zhu et al. claimed that CENPE could be a biomarker of esophageal adenocarcinoma [9]. Moreover, the overexpressed CENPE was closely related to the poor prognosis in breast cancer (BC), which indicated that CENPE could be a potential prognosis biomarker of BC [12]. Although Wang et al. determined that CENPE could promote development and metastasis through the Wnt/β-catenin signal pathway in clear cell renal cell carcinoma [13], the function of CENPE in KICH remains unclear.

Additionally, lactate dehydrogenase A (LDHA) is one of the key enzymes in glycolysis progress which have crucial effects on cancer cell growth [14]. Meanwhile, the overproduction of lactate, which depends on the function of LDHA, in glycolysis causes triggering immune escape and promotes the development of tumors [15]. It has been identified that LDHA was overexpressed in various cancers and involved in tumorigenesis and tumor growth [16]. Furthermore, Huo et al. unraveled that the LDHA-mediated glycolysis was inhibited by LINC00671 in papillary thyroid cancer cells, which contributed to the suppression of tumor cell growth and metastasis [14]. Similarly, the effect of LDHA on KICH is also not reported, while it has been found that LDHA was involved in clear cell renal cell carcinoma [17].

Thus, in this study, we tried to explore the expression, function and regulatory axis of CENPE and LDHA in the development of KICH.

## Methods

### The collection of RNA expression and clinical information

The expression of mRNA and miRNA and corresponding clinical information (age, gender, race, M stage, N stage, T stage) were obtained from the University of California Santa Cruz (UCSC) Xena database (https://xenabrowser.net/). After removing missing data, 64 KICH samples and 24 normal samples were obtained and used for subsequent analysis. Besides, mRNA and miRNA were considered not expressed if the expressions were not detected in at least 10% samples.

### The differently expressed genes (DEGs) analysis

After normalization of the raw count data with transcripts per million (TPM) method and undergoing a log2 transformation, 58,939 genes were annotated. Then, we used the R package t.test function to evaluate the significance of each gene in the tumor group and the normal group, and used the p.adjust function to calculate the false discovery rate (FDR) of each gene, and 14,565 DEGs with an absolute log2 fold change (FC) > 1 and *p*-value < 0.05 were obtained.

### Gene Ontology (GO) term, Kyoto Encyclopedia of Genes and Genomes (KEGG) pathway enrichment analysis

Then we identified the function of DEGs in KICH progression using GO and KEGG enrichment analyses. For gene set functional enrichment analysis, we used Kegg REST API (https://www.Kegg.jp/Kegg/rest/KeggAPI.html) and org.Hs.eg.db (version 3.1.0) to obtain the latest gene annotation of KEGG and GO Pathway, which was used as the background, and then mapped the genes into the background set, and used R software package clusterProfiler (version 3.14.3) for enrichment analysis. The *p* < 0.05 and FDR < 0.25 were considered statistically significant.

### The identification of hub gene

Least absolute shrinkage and selection operator (LASSO) regression was a kind of method used for selecting candidate genes which were closely related to prognosis. After we integrate the data of overall survival (OS) time, survival status and gene expression, the lasso-cox regression was performed using R software package glmnet with fivefold cross-validation (CV). After optimal lambda value was determined, hub genes with nonzero coefficients were screened out. Meanwhile, the risk scores and risk model were determined according to the gene expression value and LASSO coefficients. After the lasso-cox method, we further evaluated the prognosis value of hub genes and clinical characteristics in KICH through multivariate cox regression in SPSS 25.

### Survival analysis

The Kaplan–Meier (K–M) survival curve was used for assessing the effect of mRNA, miRNA and risk score on prognosis in KICH. Before survival analysis, the KICH samples were divided into two groups according to the optimal truncation value determined using the maxstat of the R package. Then, we evaluated the difference between the two groups in the OS time through the survfit of the R package.

### Gene set enrichment analysis (GSEA)

We explored the function of hub genes in KICH using GSEA. At first, we obtained GSEA software (version 3.0) from the website of GSEA. Then, GESA was performed by the GSEA software using a gene set database (http://www.gsea-msigdb.org/gsea/downloads.jsp) downloaded from the molecular signatures database. The *p* < 0.05 and FDR < 0.25 were considered statistically significant.

### Immune infiltrate analysis

The tumor Immune Estimation Resource (TIMER) database (https://cistrome.shinyapps.io/timer/) server is a comprehensive resource for the systematical analysis of immune infiltrates across diverse cancer types. We analyzed the association between hub genes and 6 types of immune genes, such as B cell, CD8 + T cell, CD4 + T cell, macrophage, neutrophil and dendritic cell (DC) using TIMER. *P*-values of less than 0.05 were considered statistically significant.

### The prediction of miRNA and construction of a miRNA-mRNA regulatory network

To understand the regulatory network of hub genes in KICH, miRNAs, which could target these genes, were predicted in TargetScan (https://www.targetscan.org/vert_72/) and DIANA (http://diana.imis.athena-innovation.gr/DianaTools/index.php?r=tarbase/index). TargetScan predicts targets of mRNAs by searching for the presence of conserved 8mer, 7mer, and 6mer sites that match the seed region of each mRNA, and DIANA-TarBase provides the experimentally validated interactions of miRNA and gene.

### Statistical analysis

In this study, all experiments were repeated three times. The data were analyzed using SPSS 25 and presented as mean ± standard deviation. The comparison between the two groups was evaluated by independent sample t-test, while the comparison among multiple groups was conducted by one-way analysis of variance (ANOVA) followed by post-hoc comparisons. The survival difference between the 2 groups was determined by log-rank test, and the correlation analysis was identified by the Pearson test. *p* < 0.05 was considered a significant difference.

## Results

### Identification and enrichment analysis of DEGs in the KICH based on the TCGA database

**Fig. 1:**
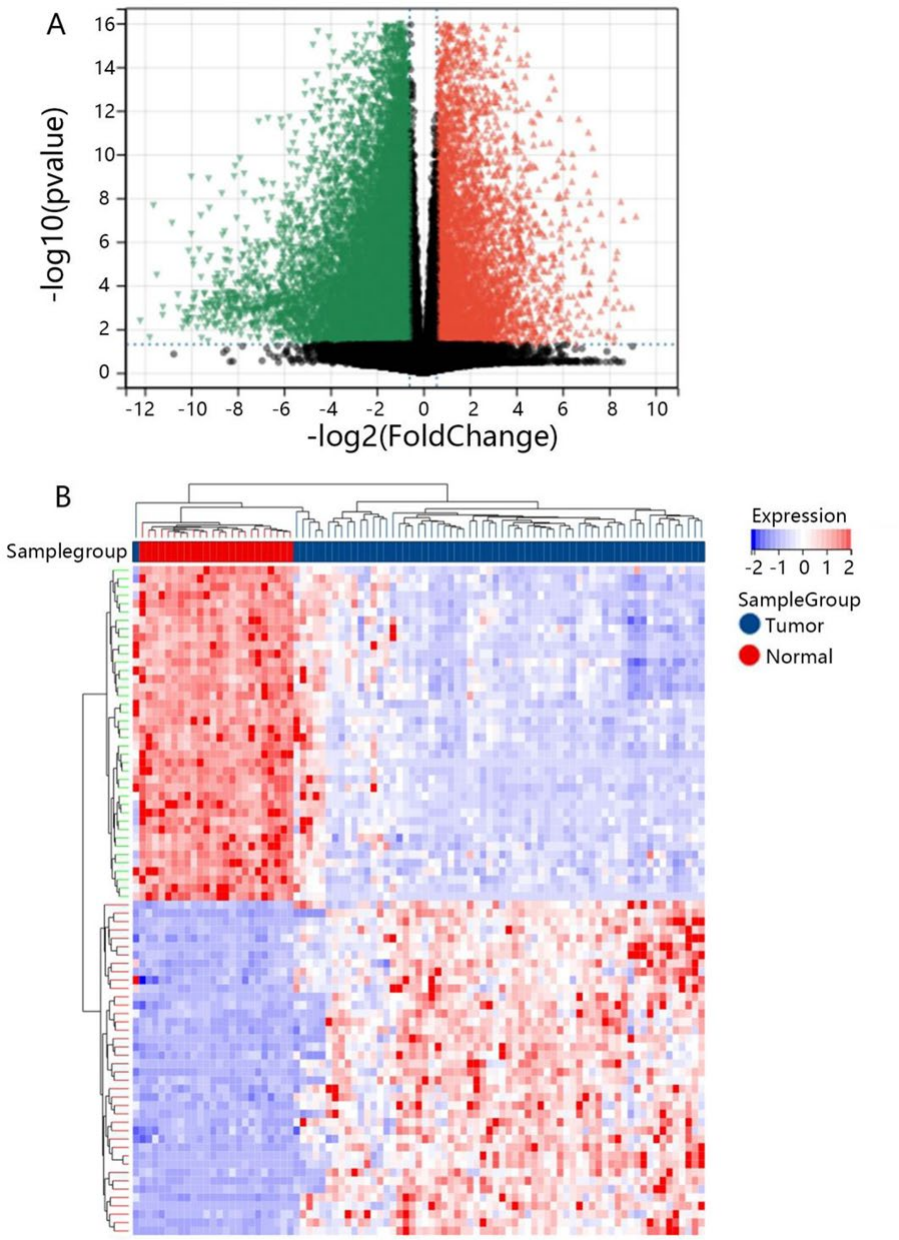
The expression profile of DEGs in KICH and normal samples. **A** The volcano plot of DEGs. The red triangle indicated upregulated genes, while the green triangle indicated downregulated genes, and the black triangle indicated genes without significantly differential expression in volcano plots. **B** The heat map of DEGs. The red dots presented upregulated genes, and the blue dots presented downregulated genes

According to the TCGA database, we found that 6162 genes were upregulated, while 13,903 genes were downregulated in KICH samples compared with normal samples (Fig. 1A). Besides, the heat maps exhibited that KICH samples can be obviously distinguished from the normal samples according to the expression of DEGs (Fig. 1B).

**Fig. 2:**
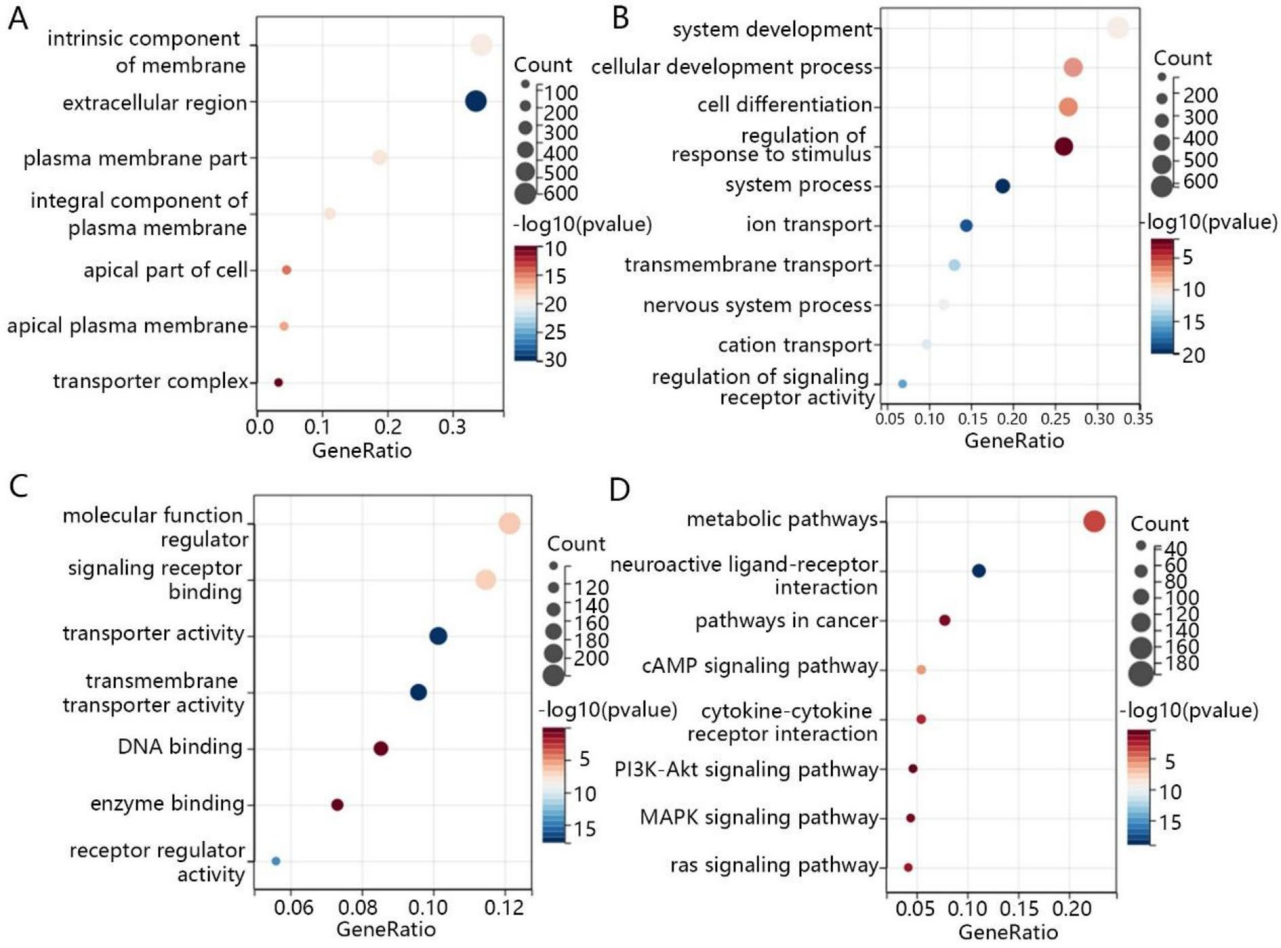
The GO and KEGG enrichment analysis of DEGs. The **A** CC, **B** BP and **C** MF of DEGs were analyzed by GO enrichment analysis. **D** The main functions of DEGs were performed by KEGG enrichment analysis. The X-axis was the value of the gene ratio and the Y-axis was the name of the corresponding terms. The size of the circles indicated the number of genes enriched in the term, and the color indicated the *P*-value

Then, GO (CC) enrichment analysis showed that DEGs were mainly enriched in intrinsic component of the membrane, extracellular region and plasma membrane part (Fig. 2A). In the biological process, the DEGs primarily play a role in system development, cellular development process and cell differentiation (Fig. 2B). Additionally, the molecular functions of DEGs were enriched in molecular function regulator, signaling receptor binding and transporter activity (Fig. 2C). The results of KEGG enrichment analysis revealed that the DEGs were mainly involved in the metabolic pathway, neuroactive ligand-receptor interaction, pathways in cancer, cyclic adenosine 3’, 5’-monophosphate (cAMP) signaling pathway, cytokine-cytokine receptor interaction, phosphoinositide 3-kinase (PI3K)—protein kinase B (AKT) signaling pathway, mitogen-activated protein kinase (MAPK) signaling pathway and ras signaling pathway (Fig. 2D).

### Establishment and verification of the risk model for KICH

**Fig. 3:**
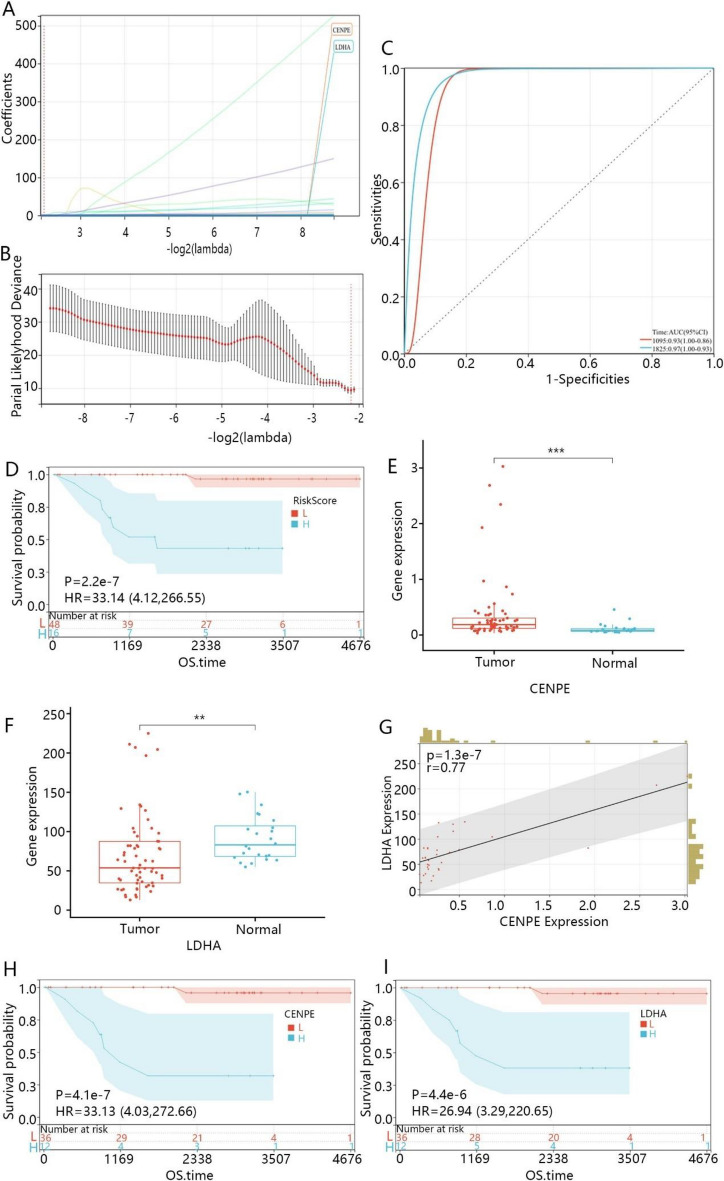
Identification and screening of the prognosis-related genes. **A** Least absolute shrinkage and selection operator (LASSO) coefficient profiles of DEGs. **B** Partial likelihood deviance for LASSO coefficient profiles. The red dots represent the partial likelihood values, the gray lines represent the standard error (SE). **C** ROC curve based on the risk model at 3 year and 5 year. **D** The K–M curve based on the risk score. **E** The expression of CENEP in tumor and normal samples. **F** The expression of LDHA in tumor and normal samples. **G** The correlation of CENPE and LDHA expressions in KICH. **H** The effect of CENPE expression level on OS time. **I** The effect of LDHA expression level on OS time. L indicated low expression of CENPE or LDHA low risk score, while H indicated high expression of CENPE or LDHA or high risk score. **p < 0.01; ***p < 0.001

Then we further selected the genes strongly related to the OS time using Lasso-regression analysis according to the DEGs expression profile. The results revealed that two DEGs with nonzero coefficients namely CENPE and LDHA were finally identified when the lambda coefficients were 0.2218 (Fig. 3A, B). Additionally, the LASSO coefficient of CNEPE and LDHA was 0.0179 and 0.0014, respectively. Moreover, we established the risk score model based on the gene coefficients of CENPE and LDHA. The risk score model was constructed as follows: RiskScore = 0.0179*CENPE + 0.0014*LDHA.

Subsequently, we generated the ROC curve of the risk model. The AUC of 3 years and 5 years were 0.93 and 0.97, respectively (Fig. 3C), which suggested the 3-year and 5-year survival rates could be favorably predicted by the risk model. The survival analysis further indicated that high risk score was related to a bad prognosis compared to the low risk score (Fig. 3D). Then, according to the mRNA expression, we found CENPE was significantly upregulated (Fig. 3E), while LDHA was downregulated in KICH samples compared to the normal samples (Fig. 3F). However, there was a positive correlation between the expression of CENPE and LDHA (Fig. 3G). Moreover, as shown in Fig. 3H, I, the high expressions of CENPE and LDHA were both related to bad prognosis in KICH samples.

**Fig. 4:**
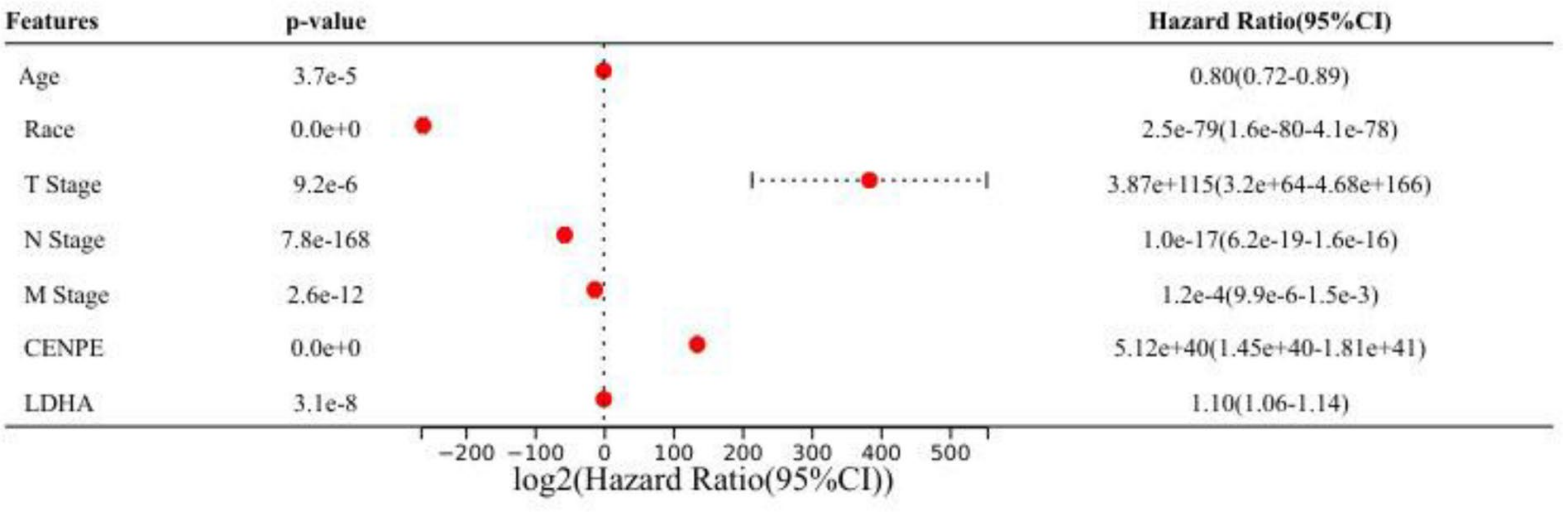
The results of multivariate cox regression analysis

Besides, the multivariate cox regression analysis revealed that age, race, T stage, M stage, N stage, CENPE expression and LDHA expression were independenly associated with OS time (Fig. 4), which indicated that the CENPE and LDHA were potential biomarkers of KICH.

### The GSEA enrichment analysis of CENPE and LDHA in KICH

**Fig. 5:**
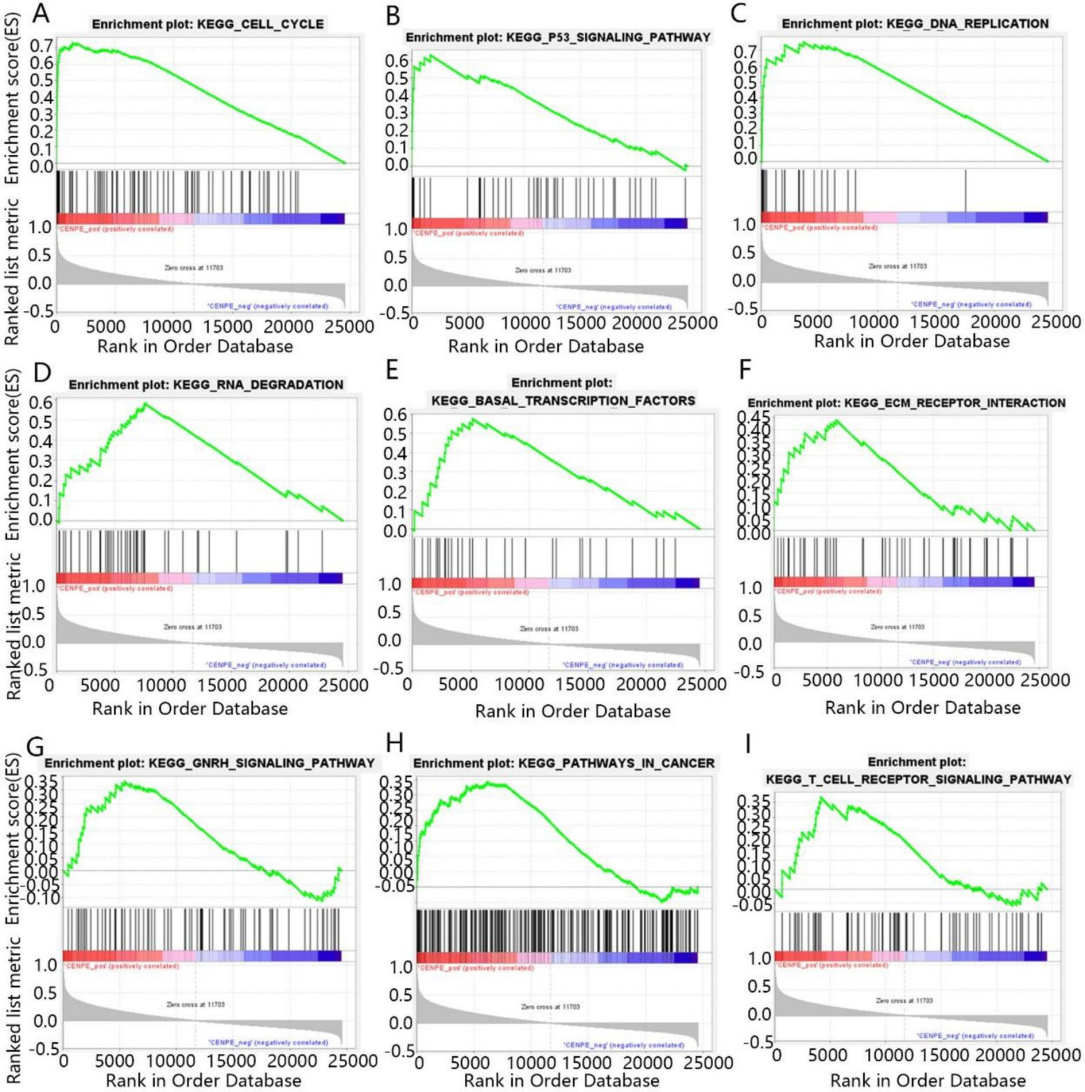
The GSEA enrichment analysis results of CENPE. **A** Cell cycle, **B** p53 signaling pathway, **C** DNA replication, **D** RNA degradation, **E** basal transcription factors, **F** receptor interaction, **G** GNRH signaling pathway, **H** pathways in cancer, **I** T cell receptor signaling pathway

**Fig. 6:**
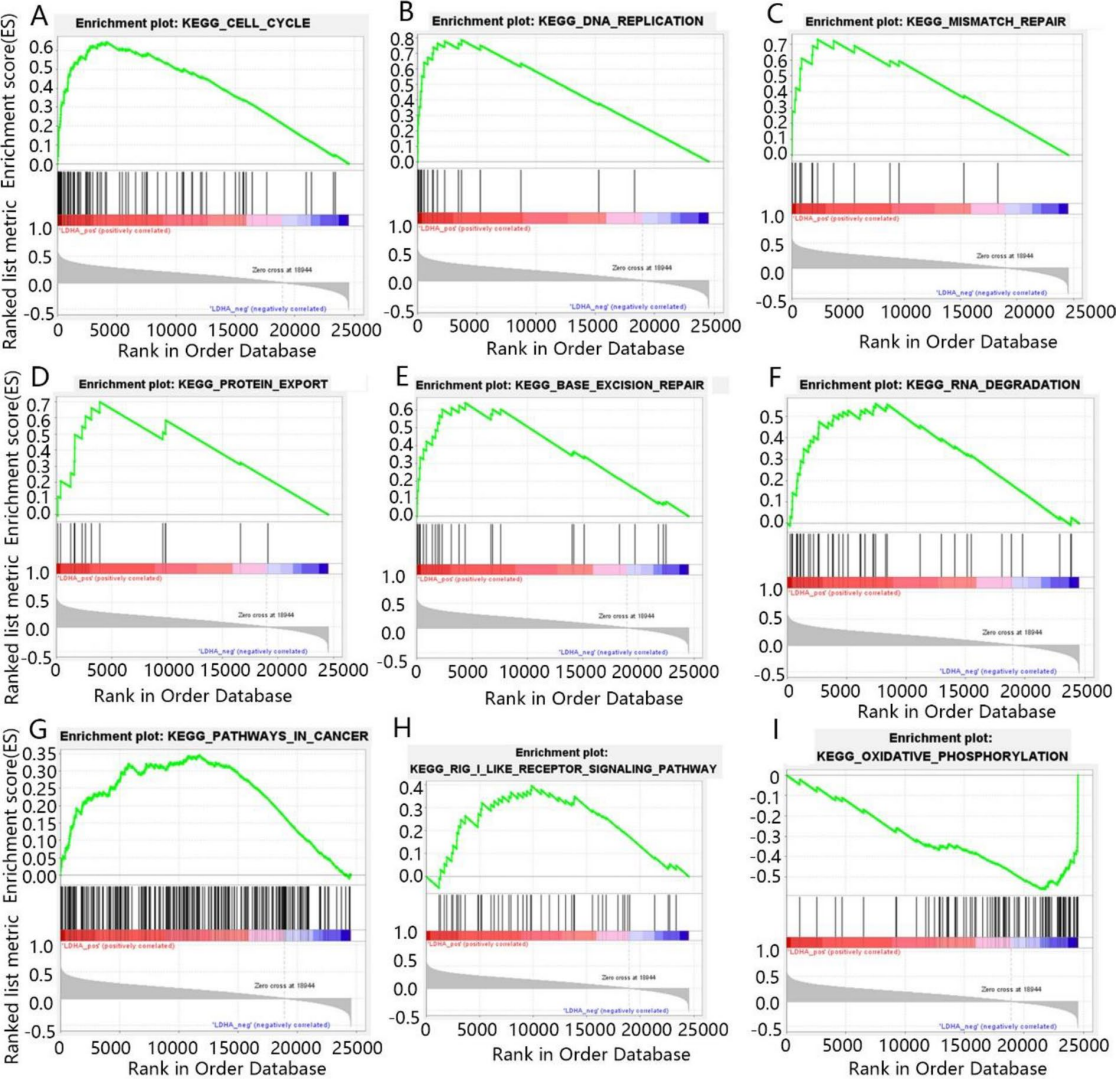
The GSEA enrichment analysis results of LDHA. **A** Cell cycle, **B** DNA replication, **C** mismatch repair, **D** protein export, **E** base excision repair, **F** RNA degradation, **G** pathways in cancer, **H** RIG-I like receptor signaling pathway, **I** oxidative phosphorylation

To further explore the role of CENPE and LDHA in the development of KICH, we analyze their function using Gene Set Enrichment Analysis (GSEA) enrichment analysis. GSEA analysis results showed that 143 pathways were positively regulated by CENPE and 35 pathways were negatively regulated by CENPE in KICH. As can be seen from Fig. 5A–I, the main pathway positively regulated by CENPE contained that cell cycle, p53 signaling pathway, DNA replication, RNA degradation, basal transcription factors, receptor interaction, GNRH signaling pathway, pathways in cancer and T cell receptor signaling pathway. In addition, LDHA promoted 134 pathways but inhibited 30 pathways in KICH. As shown in Fig. 6A–I, LDHA positively regulated the cell cycle, DNA replication, mismatch repair, protein export, base excision repair, RNA degradation, pathways in cancer and retinoic acid-inducible gene I (RIG-I) like receptor signaling pathway, but negatively regulated oxidative phosphorylation.

### The immune cell infiltration of CENPE and LDHA

**Fig. 7:**
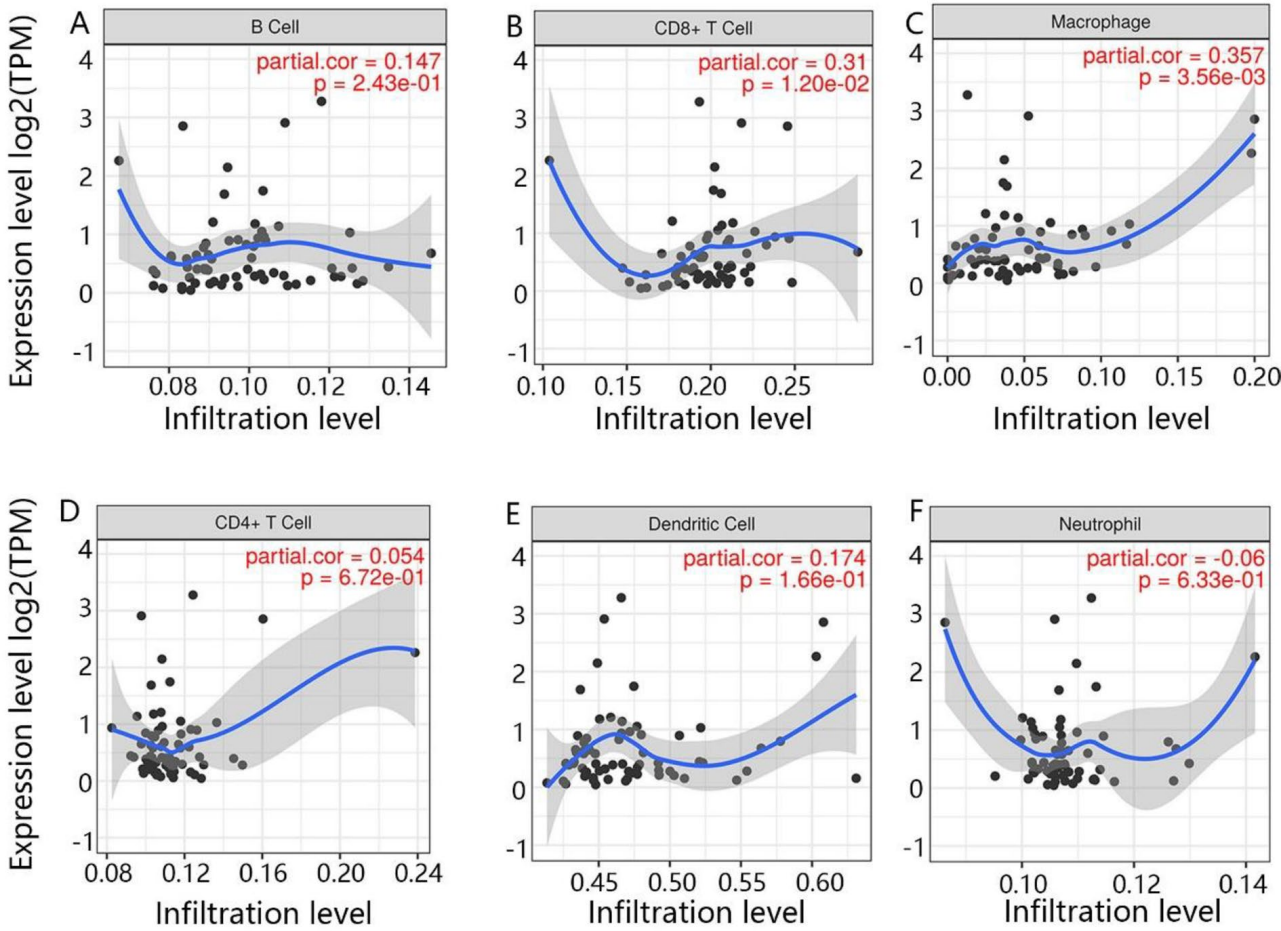
The correlation between CENPE and immune cells. **A** B cell, **B** CD8 + T cell, **C** macrophage, **D** CD4 + T cell, **E** DC, **F** neutrophil

**Fig. 8:**
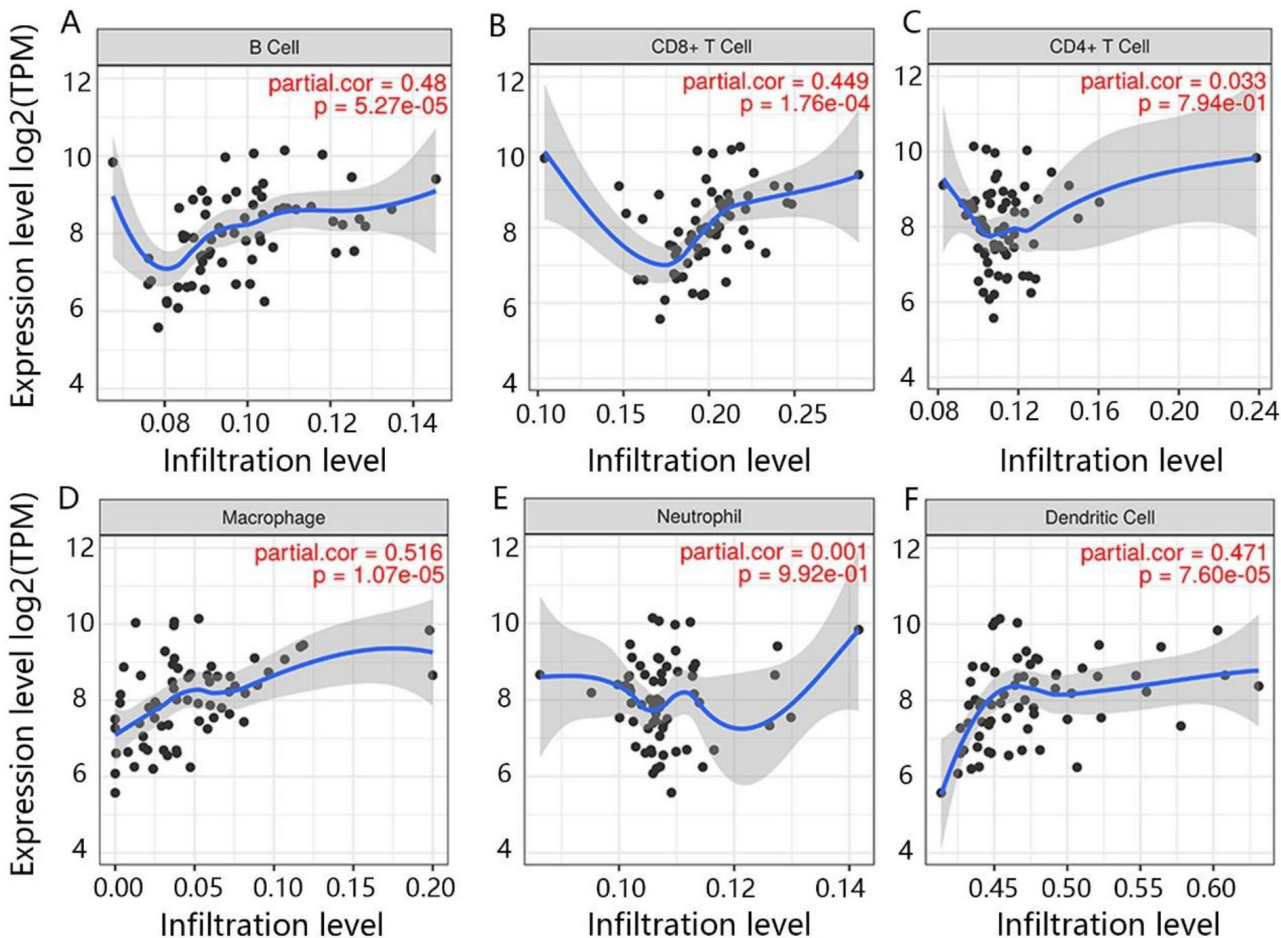
The correlation between LDHA and immune cells. **A** B cell, **B** CD8 + T cell, **C** CD4 + T cell, **D** macrophage, **E** neutrophil, **F** DC

Because some enriched pathways, including T cell receptor signaling pathway and RIG-I like receptor signaling pathway, were closely related to the immune response, we further determined the correlation between hub genes and six immune-related cells, including B cell, CD8 + T cell, CD4 + T cell, macrophage, DC and neutrophil. From Fig. 7A–F, CENPE was positively and significantly related to CD8 + T cell and macrophage. Besides, there was a positive and significant relation between LDHA and four immune cells, namely B cell, CD8 + T cell, macrophage and DC (Fig. 8A–F).

### The expression and prognosis value of hsa-miR-577 in KICH

**Fig. 9:**
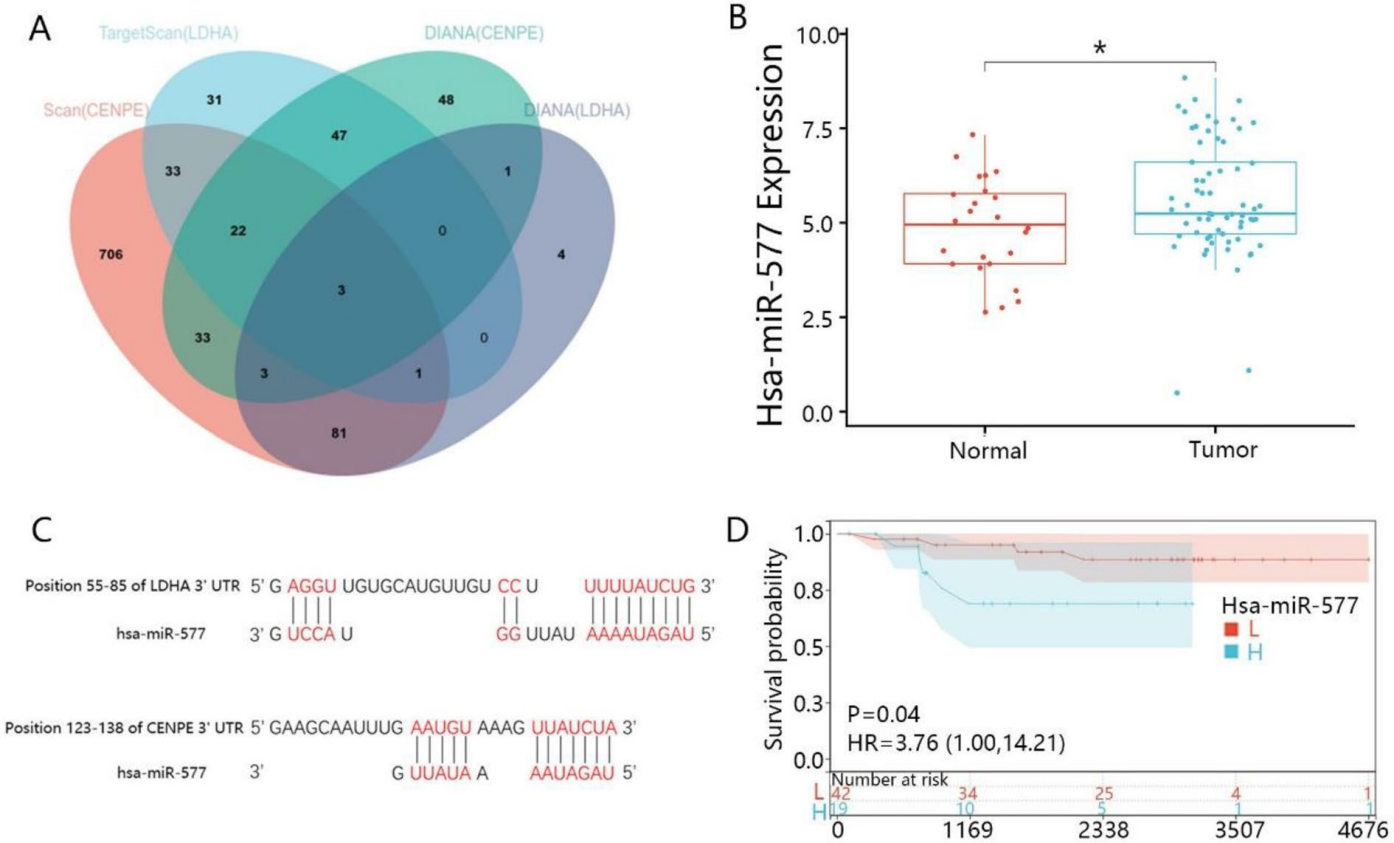
The validation, expression level and prognosis value of hsa-miR-577. **A** The miRNAs were screened out from the intersection of four databases. **B** The expression of hsa-miR-577 was based on the miRNA expression profile. **C** The combine sites between has-miR-577 and CENPE or LDHA. **D** The correlation between hsa-miR-577 expression and OS time. L indicated a low expression of hsa-miR-577, and H indicated a high expression of hsa-miR-577. *p < 0.05

To explore the regulatory pathway of CENPE and LDHA, we tried to validate the miRNA which could both target the two genes in KICH. According to the prediction of DIANA and TargetScan, we found three miRNAs in the intersection of four databases (Fig. 9A), namely hsa-miR-577, has-miR-4307 and has-miR-4470. We found that hsa-miR-577 expression was upregulated in KICH samples compared with normal samples (Fig. 9B). The other 2 miRNAs were not expressed in KICH and normal samples according to the miRNA expression profile from TCGA. Additionally, prediction results showed the has-miR-577 may bind to CENPE in the transcript position 55–85, while it may bind to LDHA in the transcript position 123–138 using DIANA (Fig. 9C). Moreover, we analyzed the prognosis value of hsa-miR-577 through the K–M curve. The results revealed that high expression of hsa-miR-577 was closely associated with a bad prognosis (Fig. 9D).

## Discussion

In this study, according to the TCGA dataset, we identified 20,065 differentially expressed genes, including 6162 upregulated genes and 13,903 downregulated genes, in KICH samples compared with normal samples. Besides, the results of GO and KEGG enrichment analysis suggested that the DEGs were involved in the development of tumors and immune [18–21]. Then, LASSO regression analysis was applied to screen out 2 hub genes, namely CENPE and LDHA. Besides, we established a risk score model based on these 2 genes, and the results of ROC curves indicated that the model could accurately predict the prognosis for patients with KICH, with the AUC of 0.93 and 0.97 for the 3-year and 5-year survival time, respectively. Furthermore, the results of the K–M plot showed that the high risk score was associated with a bad prognosis of patients diagnosed with KICH, which suggested that high expressions of CENPE and LDHA may be involved in the development of KICH.

CENPE had been regarded as a potential biomarker of diverse cancers, such as invasive ductal carcinoma and non-small cell lung cancer [11, 22]. As a mitotic cell cycle-associated gene, CENPE has an essential and positive function in the process of mitotic cytoplasmic separation, which was related to the cell cycle [9, 23], and the rapid proliferation is a common characteristic of tumor cells [24, 25]. Shi et al. certificated that the silencing CENPE led to the inhibition of cell proliferation and promotion of apoptosis in acute myeloid leukemia cells [26]. In addition, Wang et al. found that overexpressing CENPE promoted the cell viability, migration, and invasion of neuroblastoma [27]. These results suggested that the high expression of CENPE could promote the development of tumors. In our study, we found that CENPE was upregulated in KICH samples compared to the normal samples. Besides, the high expression of CENPE could independently predict a bad prognosis in KICH samples according to the K–M plot and multivariate cox regression analysis. These results indicated that CENPE was a potential biomarker of KICH with cancer promoting activity.

RCC is essentially a metabolic disease characterized by a reprogramming of energetic metabolism [28–31]. In particular the metabolic flux through glycolysis is partitioned [32–34], and mitochondrial bioenergetics and OxPhox are impaired, as well as lipid metabolism [32, 35–37]. As a kind of lactate dehydrogenase enzyme, LDHA played a vital role in glucose metabolism and many signal pathways related to cancer development [38, 39], and it has been regarded as a biomarker for prognosis of many cancers [40–42]. Additionally, the development of tumors could be modulated through regulating glucose metabolism [43]. Although oxidative phosphorylation was the preferred energy production process, cancer cells including clear cell renal carcinoma (ccRCC) usually acquired energy from glycolysis [44], and further studies proved that LDHA could also promote tumor progression through glycolysis [45]. However, in direct contrast to ccRCC which is the most common subtype of RCC, KICH have an irregular metabolic program [46]. Previous studies identified that the increased oxidative phosphorylation was observed in KICH [47]. In this study, we found that LDHA expression downregulated in KICH samples compared to the normal samples, but it was high-expressed in most cancers. In addition, the K–M plot and multivariate cox regression analysis verified that high expression of LDHA also could independently predict bad prognosis in KICH samples, which suggested that LDHA was a potential biomarker of KICH. Moreover, the GSEA results showed that LDHA negatively regulated oxidative phosphorylation. It meant that the LDHA provided the energy to promote the KICH growth through glycolysis and aggravate the KICH prognosis through oxidative phosphorylation.

In addition, RCC is one of the most immune-infiltrated tumors [48, 49]. Emerging evidence suggests that the activation of specific metabolic pathway have a role in regulating angiogenesis and inflammatory signatures [50, 51]. Features of the tumor microenvironment heavily affect disease biology and may affect responses to systemic therapy [52–55]. Previous studies demonstrated that LDHA was involved in the development of cancer through multiple signal pathways, including AKT/ mammalian target of rapamycin (mTOR), the c-Jun NH (2)-terminal kinase (JNK) and RIG-I like receptor signal pathway [56–58]. Besides, we found LDHA also promoted cancer progression through being involved in the cell cycle and DNA replication. Furthermore, RIG-I like the receptor signaling pathway was related to the immune response [59], which suggested that LDHA may be also involved in the immune response. Our results of immune infiltration analysis demonstrated that LDHA was significantly and positively related to the B cell, CD8 + T cell, macrophage and DC. Thus, combined with the expression and prognosis value of LDHA in KICH, we inferred that the downregulation of LDHA might inhibit the anti-tumor immune response and promote the initiation of KICH. Besides, the GSEA enrichment analysis revealed that the CENPE played a positive role in the cell cycle, p53 signaling pathway and DNA replication, which cloud promote tumor cell proliferation [60–62]. It was worth noting that the function of highly expressed CENPE was also enriched in the T cell receptor signaling pathway. It had been reported that high expression of CENPE promoted immune cell infiltration, especially B cells [63]. Similarly, we also found that CENPE was significantly and positively related to CD8 + T cell and macrophage. However, it seems to be against the function of CENPE as a carcinogenic factor, because the CD8 + T cell and macrophage could inhibit the development of cancer [64, 65].

It had been reported that CNEPE and LDHA could be targeted by miRNA in some cancers [66, 67]. What’s more, there was a wide regulatory spectrum between mRNA and miRNA, because the mRNA could bind to several miRNAs and the miRNA could bind to hundreds of mRNAs [68]. According to the four prediction datasets and expression datasets, we found hsa-miR-577 was the common target of CENPE and LDHA. We speculated that hsa-miR-577 may target CENPE and LDHA and influence the progression of KICH. It had been reported that hsa-miR-577 was involved in the progression of renal cancer except for KICH [69, 70]. Our study demonstrated that hsa-miR-577 was upregulated in KICH and might promote KICH progression through targeting CENPE and LDHA. The detailed regulation of hsa-miR-577 in KICH needs further investigation.

Though this study provided 2 important biomarkers for KICH, several limitations should be acknowledged. For example, the factors contributed to the LDHA downregulated in tumor samples remains unclear, and the mechanism of CENPE as a carcinogenic factor positively regulating the immune cells was not clarified. These problems might be the direction of the future research.

## Conclusions

This study identified 2 prognosis-related biomarkers in KICH, namely CENPE and LDHA. The CENPE was upregulated while LDHA was downregulated in tumor tissues. Enrichment analysis predicted that both CENPE and LDHA were involved in proliferation-related pathways such as cell cycle and DNA replication. The 2 genes were also associated with immune infiltrates, implying that they may participate in the immune response in KICH. The hsa-miR-577 was further found to be the common upstream marker of the 2 genes. Our study provided 2 important biomarkers for KICH and potential regulatory mechanism, and detailed function needs to be verified in vitro experiments.

## Data Availability

The dataset used and/or analyzed during the current study is available from the corresponding author on reasonable request.
